# Endoscopic retrograde cholangiopancreatography is not necessary in all patients with an abnormal intraoperative cholangiogram

**DOI:** 10.1002/jgh3.12984

**Published:** 2023-10-25

**Authors:** Andrawus Beany, Anandpreet S Ghataura, Shaanan T E Yong, Kee F Loo, Rajvinder Singh, Biju George, Mohamed A Chinnaratha

**Affiliations:** ^1^ Department of Gastroenterology Lyell McEwin Hospital Elizabeth Vale South Australia Australia; ^2^ Faculty of Health and Medical Sciences University of Adelaide Adelaide South Australia Australia

**Keywords:** endoscopic retrograde cholangiopancreatography, filling defect, gallstone disease, intraoperative cholangiogram, magnetic resonance cholangiopancreatography

## Introduction

Laparoscopic cholecystectomy is currently the standard of care for managing symptomatic gallstone disease, one of the most prevalent gastrointestinal disorders in the Western world. Intraoperative cholangiogram (IOC), which aids in early detection of iatrogenic bile duct injury during surgery, can also define biliary ductal configuration and allows identification of bile duct stones or obstruction. The incidence of suspected common bile duct (CBD) stones found on IOC ranges from 8 to 15%,^1^ and endoscopic retrograde cholangiopancreatography (ERCP) is routinely performed to investigate abnormal IOCs. However, ERCP is not an innocuous procedure and IOC in itself is associated with a significant false‐positive rate.^1^

The primary aim of this study was to assess the proportion of patients with positive ERCP among those with an abnormal IOC. Secondary aims included assessment of predictors of positive ERCP and ERCP‐related serious complications including death.

## Methods

A retrospective review of all ERCP procedures performed at a single tertiary hospital between June 2016 and December 2021 was conducted. Inclusion criteria were patients ≥18 years old who required ERCP for abnormal IOC findings during cholecystectomy, defined as either filling defect(s) or no contrast flow to the duodenum. Post‐cholecystectomy bile leak and cystic duct filling defects were excluded. Patient demographics, IOC and ERCP findings, time from cholecystectomy to ERCP, use of noninvasive imaging prior to ERCP, and ERCP‐related serious complications were collected. Positive ERCP was defined as either filling defect on cholangiogram or stones/sludge seen on balloon trawling. Univariate and multivariate logistic regression analyses were performed to determine the significant predictors of positive ERCP. ERCP‐related serious complications were included if patients required either readmission within 2 weeks or delayed discharge post ERCP.

## Results

Overall, 1008 ERCPs were performed during the study period, and among them, 103 (10.2%) were performed for an abnormal IOC. These patients had a median (IQR) age of 47 (31–67) years, median (IQR) time from cholecystectomy to ERCP of 3 (2–5) days, and were predominantly females (73.8%). CBD access (technical success) was achieved in 100% of the patients. ERCP was performed for a filling defect on IOC in 65 (63.1%) patients and in 38 (36.9%) patients for no contrast flow to the duodenum. Forty percent of patients had a single filling defect on IOC.

ERCP was positive in 70 (68%) patients. A quarter of patients (25.2%) underwent magnetic resonance cholangiopancreatography (MRCP) prior to ERCP, and among them, filling defect(s) was seen in 20 (19.4%) patients. Females and those with a filling defect on MRCP were significant predictors on univariate analysis, and a filling defect on MRCP was the only independent predictor of positive ERCP on multivariate analysis (Table 1). The positive predictive value (PPV) of MRCP was 92.3% compared to 68% for IOC. Five (4.9%) patients had post‐ERCP pancreatitis (two with normal cholangiogram on ERCP). There was no mortality in this cohort of patients.

**Table 1:** Odds ratio (OR) for a positive endoscopic retrograde cholangiopancreatography

Variables	Univariate analysis		Multivariate analysis	
	OR (95% CI)	*P* value	OR (95% CI)	*P* value
Age	0.99 (0.97–1.01)	0.51		
Gender (F)	**3.55 (1.11–11.3)**	**0.03**	1.22 (0.08–18.3)	0.88
Bilirubin	0.99 (0.97–1.01)	0.5		
Type of IOC abnormality^†^	0.59 (0.25–1.37)	0.22		
Number of stones in IOC^‡^	0.57 (0.17–1.85)	0.35		
Filling defect on MRCP	**9.0 (1.03–78.6)**	**0.047**	**9.1 (1.03–79.9)**	**0.047**

^†^ Filling defect or no flow to the duodenum.

^‡^ Single.

Bold values are given to highlight the statistical significance.

CI, confidence interval; IOC, intraoperative cholangiogram; MRCP, magnetic resonance cholangiopancreatography.

The findings on ERCP and IOC might be discordant in significant proportion of patients. Our results are similar to those of previous series in which approximately one‐third to one‐half of patients with an abnormal IOC have a normal postoperative ERCP.^1^, ^2^, ^3^, ^4^, ^5^ We believe that these false‐positive rates might be due to misinterpretation of the cholangiogram during the IOC (e.g. air bubbles, surrounding edema or spasm), passage of the stones prior to ERCP, or nonvisualization of stones at the time of their extraction. Abnormal blood tests, sonographic findings, and IOC findings have failed to predict choledocholithiasis or to identify patients who merit further evaluation by ERCP.^3^, ^6^ Thus, patients with a positive IOC may benefit from other studies prior to an endoscopic intervention. Endoscopic ultrasonography (EUS) was found to have a high PPV in this setting,^4^ but a noninvasive method, such as MRCP, might be favored. Comparing the findings of MRCP and ERCP, MRCP was found to have high diagnostic accuracy for bile duct stone (89.3%).^7^ Our study adds further evidence to support the role of MRCP prior to ERCP as it is an independent predictor of choledocholithiasis at ERCP in patients with suspected filling defects on IOC.

ERCP is an invasive procedure with potential to cause serious adverse events. The complication rates are as high as 15% across the studies and include pancreatitis, bleeding, perforation, cholangitis, cholecystitis, and instrument‐related complications (e.g. stent‐migration), which may often be fatal.^8^ Post‐ERCP pancreatitis is the most frequent among them, ranging between 2% and 15% of cases and accounts for substantial morbidity, occasional mortality, and increased healthcare expenditures.^9^ Interestingly, a single‐center case–control study showed a fivefold increased risk of post‐ERCP pancreatitis following a positive IOC compared with an age–sex matched cohort.^10^ In our study, similar to previous studies,^5^ post‐ERCP pancreatitis was encountered in about 5% of cases, including two patients who had a negative ERCP.

The limitations of our study are the retrospective nature of data collection, single‐center experience, and the relatively small number of subjects. The data, nevertheless, were collected very fastidiously, and ERCP reports of more than 1000 patients were thoroughly reviewed.

## Conclusions

Almost a third (32%) of ERCPs performed for an abnormal IOC in our study cohort were normal. Given the risks involved with ERCP, it may be prudent to perform MRCP prior to an ERCP as MRCP was the only independent predictor of positive ERCP.

## References

[1] Varadarajulu S , Eloubeidi MA , Wilcox CM , Hawes RH , Cotton PB . Do all patients with abnormal intraoperative cholangiogram merit endoscopic retrograde cholangiopancreatography? Surg. Endosc. 2006; 20: 801–805. 16544073 10.1007/s00464-005-0479-9

[2] Bill JG , Kushnir VM , Mullady DK *et al*. Evaluation of patients with abnormalities on intraoperative cholangiogram: time to abandon endoscopic retrograde cholangiopancreatography as the initial follow‐up study. Frontl. Gastroenterol. 2016; 7: 105–109. 10.1136/flgastro-2015-100597 PMC5369474 28839843

[3] Spinn MP , Wolf DS , Verma D , Lukens FJ . Prediction of which patients with an abnormal intraoperative cholangiogram will have a confirmed stone at ERCP. Dig. Dis. Sci. 2010; 55: 1479–1484. 19629686 10.1007/s10620-009-0894-1

[4] Luthra AK , Aggarwal V , Mishra G , Conway J , Evans JA . A prospective blinded study evaluating the role of endoscopic ultrasound before endoscopic retrograde cholangiopancreatography in the setting of “positive” intraoperative cholangiogram during cholecystectomy. Am. Surg. 2016; 82: 343–347. 27097628

[5] Lynn AP , Chong G , Thomson A . Endoscopic retrograde cholangiopancreatography in the treatment of intraoperatively demonstrated choledocholithiasis. Ann. R. Coll. Surg. Engl. 2014; 96: 45–48. 24417830 10.1308/003588414X13824511650290 PMC5137657

[6] Kaif M , Agrawal D , Sreenarasimhaiah J . Can clinical factors predict the need for intervention after a positive intraoperative cholangiogram? J. Dig. Dis. 2017; 18: 410–415. 28547873 10.1111/1751-2980.12488

[7] Ali F , Aamir N , Hassan MK , Fakhar‐E‐Alam K , Khan HU , Khan D . Comparison of MRCP and ERCP findings: a retrospective secondary data analysis. J. Pak. Med. Assoc. 2022; 72: 284–286. 35320178 10.47391/JPMA.20-721

[8] Akshintala VS , Singh A , Singh VK . Prevention and management of complications of biliary endoscopy. Gastrointest. Endosc. Clin. N. Am. 2022; 32: 397–409. 35691688 10.1016/j.giec.2022.03.001

[9] Kochar B , Akshintala VS , Afghani E *et al*. Incidence, severity, and mortality of post‐ERCP pancreatitis: a systematic review by using randomized, controlled trials. Gastrointest. Endosc. 2015; 81: 143–149.e9. 25088919 10.1016/j.gie.2014.06.045

[10] Sitaraman LM , Knotts RM , Kim J , Mahadev S , Lee DS . Increased risk of pancreatitis after endoscopic retrograde cholangiopancreatography following a positive intraoperative cholangiogram: a single‐center experience. Clin. Endosc. 2021; 54: 107–112. 32666773 10.5946/ce.2020.025 PMC7939779

